# Neglected simultaneous bilateral femoral neck fractures secondary to narcotic drug abuse treated by bilateral one-staged hemiarthroplasty: a case report

**DOI:** 10.1186/1749-799X-5-41

**Published:** 2010-06-25

**Authors:** Alireza Hootkani, Ali Moradi, Ehsan Vahedi

**Affiliations:** 1 Orthopaedics division, Emamreza Medical Center, Mashhad University of Medical Sciences, Mashhad, Iran

## Abstract

Simultaneous bilateral femoral neck fractures are extremely rare and associated with various conditions. Up to now Most cases had correlations with major trauma, repetitive minor trauma, seizure, parathyroid or renal dysfunction, anti-epileptic medications, seizure, etc. A 28-year-old addict man referred to us with a 10-year history of narcotic drug abuse and history of 8 months bilateral groin pain. He admitted with displaced bilateral femoral neck fracture. Because of long duration of this condition and osteonecrosis revealed on bone scan, one-staged bilateral hip hemiarthroplasty was done. A good function was noted after surgery to 4-month follow up. Up to now, have not be founded in the literature that a case of bilateral femoral neck fracture associated with narcotic drug abuse.

Because of negative effects of opium or smoking on bone tissues, a simple bone pain should aware us about the risk of stress or fatigue fracture.

## Background

Simultaneous bilateral femoral neck fractures are extremely rare and have been associated with high-energy trauma [1], repetitive minor trauma, abnormal anatomy [2], seizure [3-9], electrical injury, electroconvulsive therapy, primary or secondary bone diseases such as osteomalacia [10], hyperparathyroidism [11], chronic renal failure [12], or severe osteoporosis especially after corticosteroid or other corticosteroid-like drug therapy.

Different procedures have reported in the literature for treatment of bilateral femoral neck fracture include in situ fixation, open reduction and internal fixation, open fixation with valgus intertrochanteric osteotomy [13] and hemi or total hip arthroplasty in one [14] or two-staged operations.

The complications include: non-union, delayed union and shortening. Femoral head osteonecrosis and coxa vara can be avoided with correct treatment.

In this report, we present a case of bilateral femoral neck fractures with prolonged history of narcotic drug abuse, treated by bilateral hemiarthroplasty. We did not found any previously recognized underlying risk factors for stress fracture.

## Case presentation

**Figure 1:**
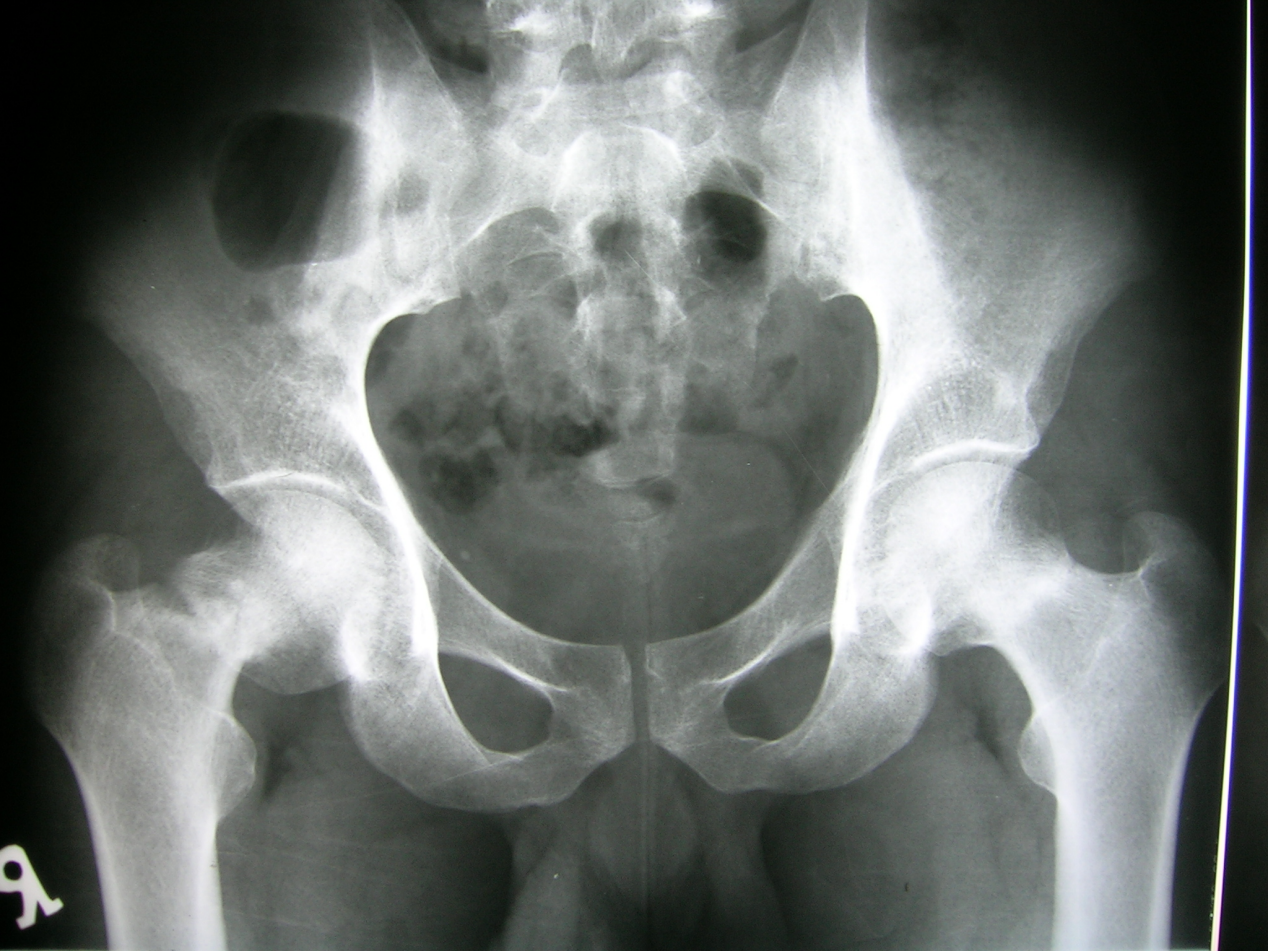
**Radiograph taken 4 months before operation demonstrates displaced left femoral neck stress fracture and non-displaced right femoral neck stress fracture**.

A 28-yr-old addict man was referred to our institution with an 8 month history of chronic pain in both groins aggravated by weight bearing sometimes keeping him awake at night. He had previously normal activities without specific sport or job program. By disease onset, His activities were restricted because of the pain and decreased range of motion. About 4 months before operation, the patient was visited by a physician who took a plain pelvic radiograph which revealed displaced left and non-displaced right femoral neck stress fracture (Fig-1).

**Figure 2:**
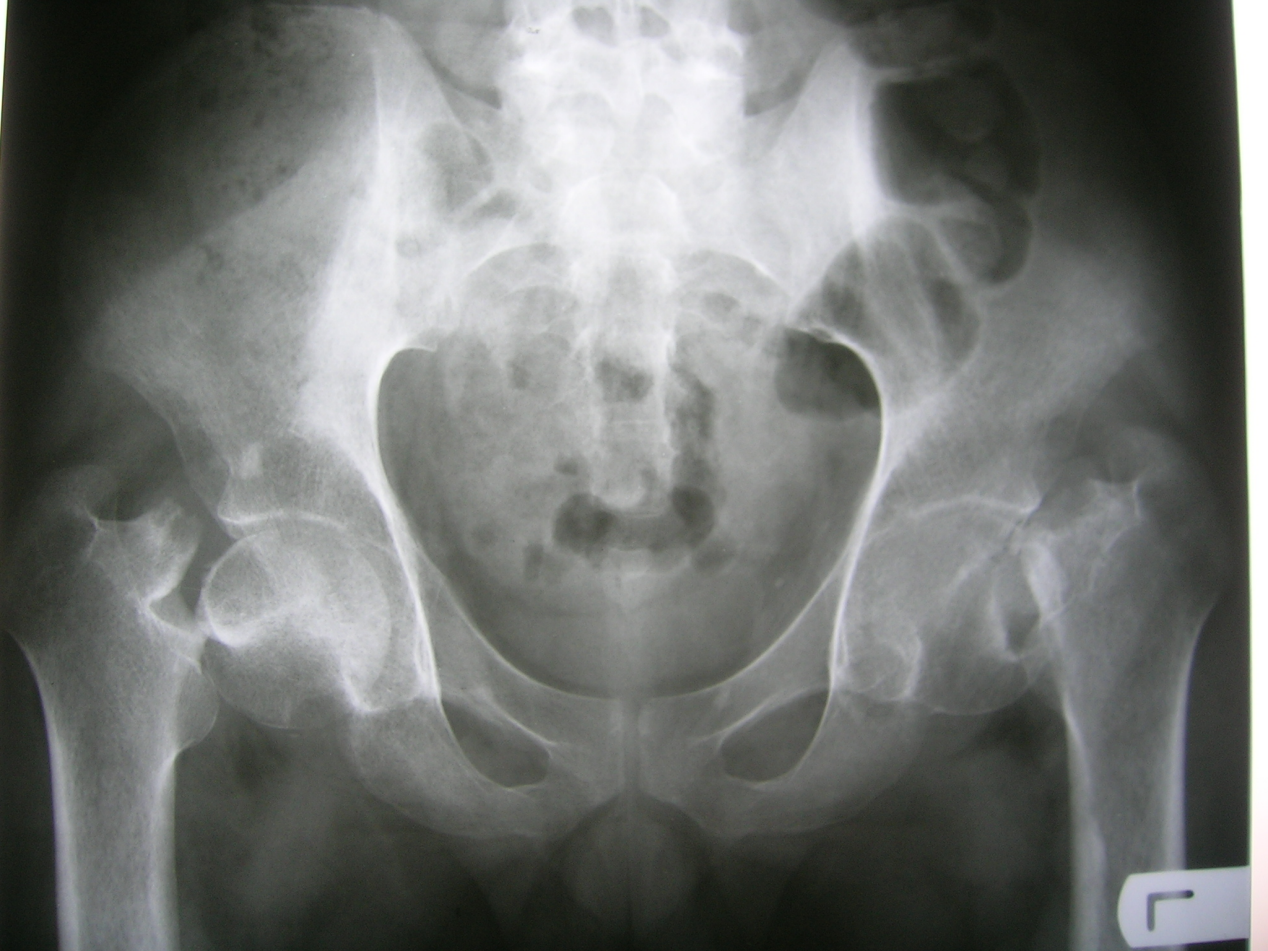
**Radiograph taken before operation demonstrates displaced bilateral femoral neck stress fractures**.

After 8-month, because of low socio-economic state and not appropriate follow-up by him, finally, the patient was referred to us with displaced bilateral hip fractures (Fig-2).

On examination, his weight was 65 kilograms and both active and passive motions were painful and restricted. Active straight leg raise was not possible on either side. No limb-length discrepancy was observed because of symmetrical nature of the deformity.

There was no evidence of any underlying metabolic or endocrine disease affecting the bone strength in physical examination or family history.

In laboratory tests, CBC, ESR, alkaline phosphatase, serum calcium, phosphorus and serum total protein levels were normal. Normal Parathyroid, thyroid, renal, gonadal and liver function tests were detected. 24-hour urinary excretion for calcium and phosphorus was also within normal ranges.

Because of long period of immobilization and lesser activities, which decreased bone density, we did not consider bone densitometry for him.

**Figure 3:**
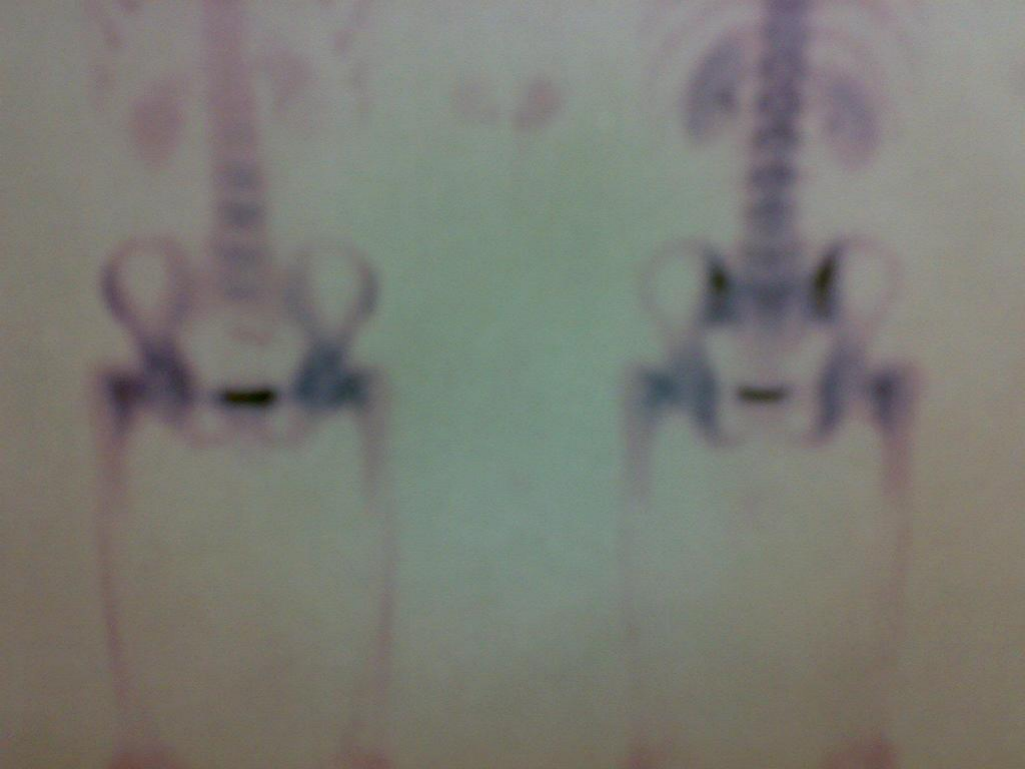
**Skeletal bone scan with TC-99m showed no other abnormal findings except bilateral femoral head osteonecrosis and bilateral femoral neck fracture**.

Skeletal bone scan with TC-99m showed no other abnormal findings except bilateral femoral head osteonecrosis and bilateral femoral neck fracture (Fig-3).

**Figure 4:**
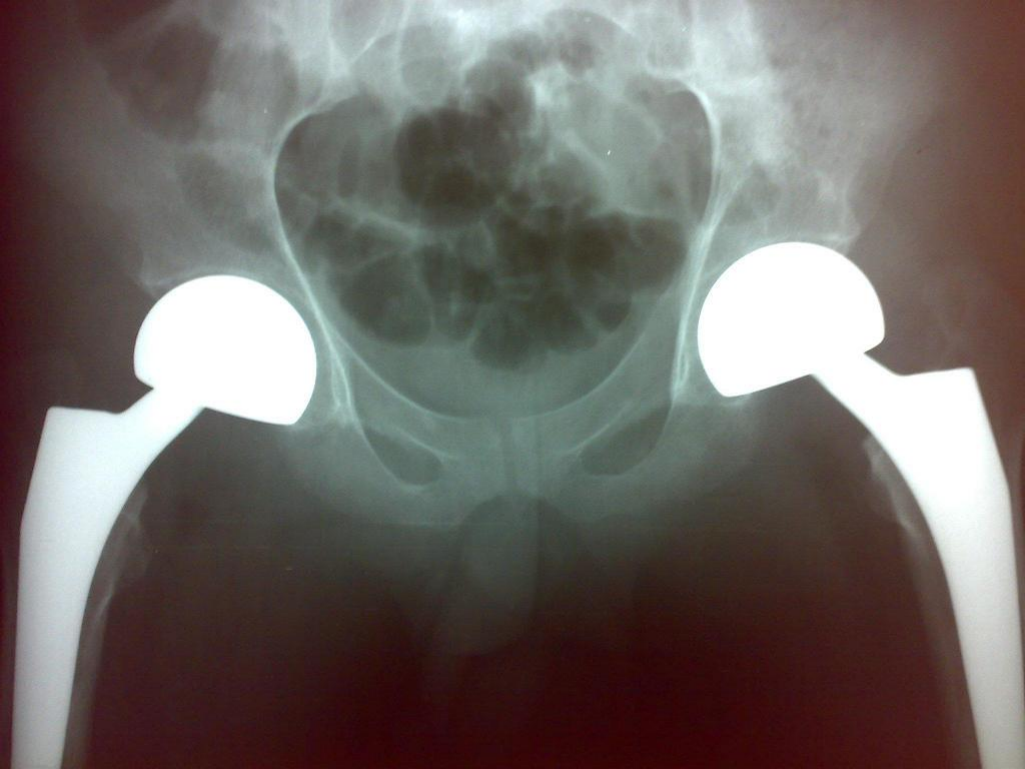
**Radiograph taken after operation demonstrates replacement of left and right hip with bipolar prosthesis**.

Surgery was performed using general anesthesia with the patient lateral on a conventional operating table for both right and left hips at one section choosing posterolateral minimal incision approach. The cartilage of the acetabulum was intact and heads of the femurs were decided to be replaced with bipolar prosthesis (Fig-4). During operation, it was noted that bone ends at the fracture site was extremely sclerotic and difficult to penetrate with instruments. A biopsy showed no evidence of metabolic bone disease and osteonecrosis was reported. The postoperative period was uneventful. Quadriceps exercises and pain free movement of both hips were permitted from the first post operative day. Then, the patient was permitted to bear weight.

The patient had normal range of motion in both hips and was capable of doing daily activities after 24 weeks of follow up.

## Discussion

Hip Stress fractures of most frequently involve the medial aspect of femoral neck. They are termed fatigue fracture in patients who have normal bone but are exposed to excessive mechanical stress, such as many seen in athletes and army recruits, and they are termed insufficiency fractures in patients who have abnormal bone subjected to normal stress.

The remarkable characteristics of this case include simultaneous, bilateral, displaced fractures with no evidence of underlying pathologic factor; suspicious relationship with narcotic drug abuse and simultaneous operation of both hips in one section with minimal incision approach.

In this case, stress fractures of femoral neck were probably related to 10 year history of drug abuse. By further abuse, the pain decreased and continuity of activities aggravated the fracture and ultimately complete bilateral femoral neck fracture occurred.

Many procedures have been reported in the literature for treatment of bilateral femoral neck fractures. These include lag screw or dynamic hip screw fixation [6-8,15,16], pedicle bone grafting [10], and a combination of these. In this case, because of the complete displacement and osteonecrosis and high risk of nonunion (23%-43%) [17-19], we decided to replace the femoral head instead of open reduction and internal fixation, and young age of the patient did not change our decision.

To our knowledge, this is the first report in the English literature of bilateral neglected displaced femoral neck fractures in which the only predisposing factor was narcotic drug abuse.

## Conclusions

The negative effects of smoking or opium on bone tissues or bone healing processes were well recognized. Also, narcotic agents diminish signs and symptoms of any painful condition affecting the bones. Because of various risk factors predisposing many people to development of stress fractures, we suggest that each patient who complaints of prolonged bone pain and has positive history of opium abuse, should be considered an important underlying bone disease such as stress fracture until proven otherwise.

## Consent

Written informed consent was obtained from the patient for publication of this case report and any accompanying images. A copy of written consent is available for review by the editor-in-chief of this journal.

## Competing interests

The authors declare that they have no competing interests.

## Authors' contributions

AH performed part of the literature review, examined the patient and operated him. Also he had given final approval of the version to be published and generally supervised this report. AM also performed part of literature review, participated in operation and conceived the idea of present study and case report presentation. EV contributes in literature review, analyzed the data and involved in editing the manuscript. All authors have read and approved the final manuscript.
